# Arsenic Elevated Groundwater Irrigation: Farmers’ Perception of Rice and Vegetable Contamination in a Naturally Arsenic Endemic Area

**DOI:** 10.3390/ijerph20064989

**Published:** 2023-03-12

**Authors:** Md Rokonuzzaman, Zhihong Ye, Chuan Wu, Wai-Chin Li

**Affiliations:** 1 Department of Science and Environmental Studies, The Education University of Hong Kong, Tai Po, Hong Kong SAR 999077, China; s1129843@s.eduhk.hk (M.R.);; 2 Department of Agricultural Extension Education, Sylhet Agricultural University, Sylhet 3100, Bangladesh; 3 School of Life Sciences, Sun Yat-sen University, Guangzhou 510006, China; 4 School of Metallurgy and Environment, Central South University, Changsha 410083, China

**Keywords:** arsenic, groundwater, perception, rice and vegetables, scalp hair

## Abstract

Arsenic (As) in groundwater and its accumulation in agricultural produces has caused serious threats to human health. The majority of current research on As mainly focuses on the technical aspects while bypassing the social perspectives. Farmers are the prime stakeholders as well as executors of agricultural strategies, and their adaptation largely depends on how they perceive the risk for which a mitigation strategy is proposed. This study aims to explore how rice and vegetable farmers perceive As accumulation in their rice and vegetables as well as explore current crop- and body-loading status, the subsequent health consequences of As, and alleviation possibilities with mitigation strategies and to investigate if there is an association between their socioeconomic status and their level of perception. Results reveal that one-fourth of the farmers gave a positive message regarding the As-contamination scenario in rice and vegetables. Although 10 farmers’ socioeconomic characteristics were positively significant, distinctive emphasis should be given to five predictor variables explaining 88% variances: knowledge, direct participation in farming, information sources used, participant education, and organizational participation. Path analysis depicts that direct participation in farming presents the highest positive total effect (0.855) and direct effect (0.503), whereas information sources show the highest positive indirect effect (0.624). The mean As content in all five locations was statistically significant at the 5%, 5%, 0.1%, 1%, and 1% probability levels in scalp hairs, rice, vegetables, soils, and irrigation water, respectively. The first principal component (PC1) explains 92.5% of the variation. Significant variations were primarily explained by As levels in irrigation water, rice grain, and soil. Farmers’ perception is far behind the actual field status of As level and its transfer. Therefore, intensified priorities should be administered on the farmers’ characteristics contributing to variances in perception. The findings can be utilized for policy formulation in all As-endemic nations. More multidisciplinary research can be undertaken on farmers’ attitude towards adopting As-mitigation techniques, with a focus on the socioeconomic position found to influence farmers’ perceptions.

## 1. Introduction

Arsenic (As) poisoning is a significant concern in 108 countries, where it affects more than 230 million people primarily through food and drinking water [1]. In regions where people are using As-free safe drinking water, some of them are still suffering from arsenic-induced diseases including arsenicosis, cancers, hyperkeratosis, restrictive lung diseases, and ischemic heart diseases [2,3]. Groundwater As exceeding the permissible limit set by the WHO (<10 μg/L) and the FAO (100 μg/L) for irrigation and its application for rice and vegetable production poses a potential health concern worldwide [3,4,5,6]. Growing rice and vegetables with groundwater contaminated with As can massively increase daily dietary As intake [1]. While more than 100 countries worldwide are involved in the rice-growing practice, 90% of the world’s rice is grown in just 14 Asian countries, with groundwater serving as the primary irrigation water supply in most [7,8,9]. This problem is even more significant in the top five rice-producing countries such as China, India, Bangladesh, Vietnam, and Indonesia [10,11,12,13]. Like rice, half of the value of vegetable exports worldwide comes from Asia (FAO/WITS, 2017). Therefore, these crops contributed to a higher risk of As poisoning from food consumption to both the As-endemic and -nonendemic populations, regardless of where they live. Several research studies have already proved that the consumption of rice and vegetables cultivated with As-elevated groundwater is a potential contributor to the human body globally [14,15,16,17].

The Meghna River basin, the eastern and northern region of the active deltaic plain of the southern coast, and the old deltaic plain of southwestern Bangladesh have all experienced high As occurrences; however, pollution is less severe in the southeastern and northwestern part of Bangladesh [18]. People living in high-risk areas knew what to look for in terms of warning signs of As poisoning and ailments induced by drinking As-contaminated groundwater [18]. Despite the fact that most of the people in the endemic area of Bangladesh opted for As-safe drinking, still, As-related ailments are prevailing there [2]. Consumption of rice and vegetables grown with As-elevated groundwater is supposed to cause this health concern [16,17]. To date, the majority of scientific interest has been devoted to determining the sources and causes of arsenic contamination and inventing cost-effective methods for removing arsenic from irrigation water [18,19]. While determining the source of the contamination and developing technologies to remediate As from groundwater are critical in combatting the problem, research efforts should go far beyond these efforts to ensure the sustainability of the technologies engaging farmers’ perspectives [18,19,20,21]. Several researchers have investigated heavy metals from the social and economic viewpoints. Thewys et al. [22] investigated the economic feasibility of employing maize as an energy crop in phytoremediation of heavy metal-contaminated agricultural areas. In heavy metal-contaminated locations, fallow compensation schemes were examined by Xie et al. [23] (2017) and Yu et al. [24] for the relevant government departments to create heavy metal management and control policies to serve as references. As Oskamp [25] said, “When thinking about environmental problems, we must recognize that they are not just technological problems that need to be solved by engineering, physical, and chemical means, but that the social sciences play a crucial role in these problems”.

To date, all efforts to minimize arsenic pollution have been made without adequate grassroots knowledge bases regarding the prime stakeholders, that is, the farmers’ perception of As accumulation in rice and vegetables due to As-contaminated groundwater irrigation. A recent scenario demonstrated that the farmers’ cooperation seriously affected implementation of the “policy of remediation during fallow (PRF)” to tackle soil fertility deterioration due to heavy metal pollution by the government of China, which finally saw the light after the survey of farmers’ perspectives and associated recommendations [9]. Therefore, a comprehensive approach for evaluating As perception is essential for establishing research priorities, ensuring development strategies, and designing pertinent stakeholder engagement to combat As-induced concerns.

This research aims to explore how rice and vegetable farmers in Bangladesh perceive As accumulation in their rice and vegetables as well as the subsequent health consequences of AS and the possibility of alleviation using mitigation strategies and to investigate if there is an association between farmers’ socioeconomic status and their level of perception. Therefore, the underlying hypothesis is that there is no relationship between farmers’ perception of As-contaminated groundwater irrigation for rice and vegetable cultivation and their socioeconomic characteristics. This endeavor also attempts to compare farmers’ perception based on the actual field-based As-accumulation data. The study’s findings should be useful in pinpointing particular socioeconomic and demographic features where further steps need to be intensified for sustainable As mitigation and farmers’ adoption of the same based on the actual As status of irrigation water and its subsequent transfer to rice and vegetables. The purpose of this study is not to investigate whether farmers had heard of the As pollution problem in general but instead to explore if they are knowledgeable and have the correct perception about crucial aspects of arsenic poisoning, such as the source of As in rice and vegetables, As symptoms, diseases caused by poisoning, and how to prevent and mitigate As poisoning.

## 2. Material and Methods

### 2.1. Study Area

The high groundwater contamination in the district of Chandpur in Bangladesh’s southeast has made it a well-known As-endemic zone [26]. Chakraborti et al. [27] found 100 to 1318 g/L As, while Jakaria et al. [28] and Mishra et al. [29] found that 80–90% of the tube wells in the area have As concentrations above 50 μg/L. According to estimates, more than 90% of the locals in this area rely on water from tube wells for both drinking and irrigation needs [26,28]. Hajiganj, Chandpur Sadar (Sadar), Faridganj, Matlab North, and Kachua, five of the most well-known and heavily As-elevated *upazilas* (subdistricts) in Chandpur, have been chosen as the study region (more information on the research site is presented in Appendix A.1).

### 2.2. Collection of Data

As Morse and Niehaus [30] note, whether a quantitative or qualitative methodology is adopted, sampling procedures are designed to maximize efficiency and validity. Nonetheless, sampling must be compatible with the objectives and assumptions inherent in either approach. Choosing settings, groups, or individuals to represent a sample in 2 or more stages while ensuring that each step reflects participant purposive sampling is known as multistage purposive sampling. Therefore, multistage purposive sampling entails picking a sample in 2 or more phases. Unlike multistage purposeful random sampling and random purposeful sampling, however, all stages include purposive sampling. Multistage purposeful sampling is distinct from mixed purposeful sampling in that it is always sequential, whereas the latter generally comprises contemporaneous sampling in which one sample is not a subset of other samples [31]. Multistage purposive sampling has been applied to select study locations and the respondents. Purposive sampling has the advantage of allowing researchers to gain a better understanding of the study’s research problems and study sites [32]. Furthermore, a necessary step in this process is identifying and choosing the right people or groups who are remarkably experienced or knowledgeable regarding the subject matter [33]. In addition to experience and knowledge, Bernard [34] and Spradley [35] note the significance of willingness, the availability to participate, and the ability to communicate experience and opinions in an articulate, reflective, and expressive manner.

In the present study as a part of the multistage purposive sampling strategy, in the first step the Chandpur district was chosen because according to the literature, it has a high level of As in its groundwater. During the second step, 5 of the subdistricts were selected based on the literature and information from agriculture offices. In the third stage, 40 farmers from each of the subdistricts (200 farmers total) were selected who met specific criteria. These farmers were required to, among other things, produce rice and/or vegetables with groundwater irrigation, consume their own field-produced rice and vegetables, be willing to participate in the study voluntarily, drink As-safe water, and be willing to donate scalp hairs to determine As content for another associated study. Selection of the farmers was accomplished with the assistance of the agriculture officer, subassistant agriculture officers (SAAOs), and leaders of the local farmer communities. The data were collected by administering an interview schedule that had been designed based on focus group discussions (FGD) and key informant interviews (KII), and the schedule was finalized following judge rating.

### 2.3. Samples Collection and Preparation

Ten farmers were purposively selected from each location to collect their scalp hair samples, 4 subsamples of vegetables, rice, soils, and irrigation water were collected from their fields to construct composite samples [36] (Appendix A.2 includes information on the plant species and the procedure for collecting samples.) Rice samples were separated from the chaff. After being cleaned for 5 min in running water and rinsed twice with deionized water, the vegetable samples were patted dry with filter paper and then dried in an oven at 60 °C for 24 h [37]. Prior to chemical analysis, the vegetable and rice samples were ground using a carnelian mortar. Male farmers in the area often have regular haircuts, making it possible to measure As concentrations in hairs from recent exposure, while women’s long hair is better suited to chronicling more extended periods of exposure; therefore, only samples from male farmers’ scalps were taken using stainless-steel scissors [38]. To standardize As levels across the scalp, 1 g of hair was obtained from each scalp site on the same person [39,40]. Aluminum foil-wrapped samples were transported to the laboratory and stored at −20 °C in zip lock bags pending chemical analysis [41]. To get rid of any debris that might have been stuck to the sample, the samples were double-rinsed with 5 mL of deionized water and methanol [42].

### 2.4. Analytical Framework

#### 2.4.1. Farmers’ Socioeconomic Characteristics

With great care, a structured interview schedule was constructed and translated into Bangla to facilitate information gathering from native speakers [20]. The data were collected through face-to-face interviews from June 2019 to August 2020. In order to characterize the socioeconomic backgrounds of farmers, 16 variables were assessed: family education, participant education, farm size, knowledge, annual income, family size, information sources, direct participation in farming, agricultural credit use, cosmopoliteness, opinionatedness, innovativeness, risk orientation, farm power and machinery (FPM), and organizational participation.

The number of years from a farmer’s birth to the interview was used to compute his age and was rounded to the nearest whole number. Regarding farmers’ education, a score of 1 was assigned for each class passed. In order to calculate family education, the overall score on education was recorded and then divided by the ‘effective family size’ [43]. The ‘effective family size’ was calculated by subtracting the number of children under the age of 4 from the total number of family members. The following formula was used to generate the index of family education, which was used to quantify family education:

Index of Family Education = Total educational score/Effective family size

Family size was determined as the total number of individual farmers’ family members. Direct participation in farming was measured by how a farmer performs agricultural work by himself rather than with others. An individual farmer could obtain a score of 0 to 3 for each agricultural operation. The score of the participants could range from 0 to 18, where 0 indicates ‘no direct participation’ and 18 indicates ‘high direct participation’ in farming. A 5-point scale checking any of the responses ‘most often,’ ‘often,’ ‘sometimes,’ ‘rarely,’ and never with scores of 4, 3, 2, 1, and 0, respectively, were provided against each item to measure the degree to which the farmers used information sources. The total rank score for each item was obtained by multiplying the frequencies with the respective weights and adding them up. Farm size was computed using the following formula and expressed in hectare (ha):

Farm size = A + {1/2(B + C) + E + F} − D

where A = land used for own farming; B = land given to others on *borga*; C = land appropriation from others on *borga*; D = land given to others on lease; E = land taken from others on lease; and F = homestead area.

Income from farming and other ethically right sources on a regular basis over a year was regarded as annual income and is expressed in 1000 takas. Similarly, agricultural credit use was expressed in 1000 takas. An adapted version of the Subramoniam [44] scale was used to assess social/organizational participation. The scale comprised 10 statements that indicated a respondent’s involvement with organizations both within and outside his living community [20]. The score given for no membership = 0, membership in one organization = 1, and office bearer in one organization = 2. Accordingly, attending meetings ‘never,’ ‘occasionally,’ and ‘regularly’ received 1, 2, and 3 points, respectively. To obtain a respondents’ final scores, the scores obtained as a member or office bearer were multiplied with the score received for attendance at meetings. The cosmopoliteness item was predicated on an individual’s orientation outside of his social structure. A 6-item (4-point scale) statement was prepared for this purpose. Each participant was required to mention how many times he traveled to each of the 6 places with the frequency of visit, such as ‘often,’ ‘occasionally,’ ‘rarely,’ and ‘never,’ and weights assigned to these responses were 3, 2, 1, and 0, respectively. A respondent’s score of cosmopoliteness was determined by summing the points allotted for the 6 different types of locations he has visited. Opinionatedness of a farmer was measured through a 4-item scale prepared for the study. Scores of 3, 2, 1, and 0 were assigned for high, medium, low, and no opinionatedness, respectively. A respondent’s innovativeness was assessed based on the relative earliness in adopting new ideas [45]; the present study included 13 improved ideas on As-reducing agricultural practices. Scores were provided based on how long it took a farmer to adopt each technique: 5 = within one year, 4 = within two years, 3 = within three years, 2 = within four years, and 1 = within five years, and 0 = do not use. A farmer’s innovativeness score was calculated by aggregating his scores for all 13 improved agricultural techniques. Risk orientation was assessed using a scale modified from Samantha ‘s [46] scale. The scale comprised 10 statements, 4 positive and the rest negative, based on Edwards’s [47] screening guidelines. Respondents’ opinions were recorded using a 5-point Likert scale [48] with positive statements assigned values of 5, 4, 3, 2, and 1 and negative statements assigned values of 1, 2, 3, 4, and 5 representing ‘strongly agree,’ ‘agree,’ ‘undecided, ‘disagree,’ and ‘strongly disagree,’ respectively. For calculating the ownership score of farm power and machinery (FPM), 7 items of farming and irrigation management tools were selected, and scores for possession of tools were assigned as follows: country plow = 1, hand sprayer = 2, rice weeder = 1, shallow tube well (STW) (joint ownership) = 3, power tiller = 4, shallow tube well (STW) (single ownership) = 4, and harvester = 4. The number of tools was multiplied by the assigned score to obtain the final score. Farmers’ knowledge was assessed based on the method used by Paul [18] with little modification. A composite score was computed based on each participant’s responses to 11 questions (into 6 groups) about the source, symptoms, and As-induced diseases as well as potential preventive approaches and remedies to the arsenic-accumulation problem in crops. One focus group discussion (FGD) was held in each upazila to establish the scores for anticipated answers consisting of farmer leaders, available rice and vegetable growers, and agriculture officers. The participants’ recommendations were considered to provide various points for correct answers and zero for incorrect ones.

#### 2.4.2. Farmers’ Perception Assessment

According to Hodgetts [49], no 2 people will have the same perception of life, and no 2 people will see things in the same way. For recording farmers’ perception, appropriate statements were prepared with the cooperation of researchers, farmer leaders, available rice and vegetable growers, and the agriculture officer and validated with data from a field survey [20]. After subjecting these statements to judges’ rating [50], the interview schedule contained 43 statements in 7 categories and was administered to the respondents for expressing their perceptions on the use of As-contaminated or safe water for rice and vegetable production. To avoid acquiescence, that is, the propensity of participants to agree or disagree with statements irrespective of the item content, the interview schedule was constructed with both negative and positive statements. According to Schweizer et al. [51], using negative and positive statements when replying to questions helps avoid phrasing problems and responder personal bias. However, the statements were rated on a 6-point Likert scale [48] where ‘strongly agree,’ ‘agree,’ ‘undecided, ‘disagree,’ and ‘strongly disagree’ were scored with 1, 2, 3, 4, and 5, respectively for negative statements and 5, 4, 3, 2, and 1 for positive statements, respectively.

#### 2.4.3. Sample Analysis and Quality Control

Atomic fluorescence spectrophotometry (AF-610A, manufactured in Beijing, China), was used to measure the content of As in vegetables, grains, soil, and water by adopting the protocol followed by Huang et al. [52]. Exactly 0.25 g of soil was weighed, and a few drops of deionized water were added to a 100 mL Erlenmeyer flask to quantify soil total As. The soil was mixed with 2 mL of concentrated HClO_4_, 5 mL of HNO_3_, and 6 mL of HCl. Over the course of about 1.5 h, the reaction died down, while the digestion continued at 150 degrees Celsius in the flask covered with a small glass filter heated with an electric heater. A 50 A volumetric flask (50 mL) was used to combine 5 mL of sulfourea solution (50 g/L) with the digest, and the remaining volume was filled with double-deionized water. Not more than 0.5g of rice grain or 0.5g of vegetables (0.5mm) was placed in an Erlenmeyer flask (100 mL) with 1.25 mL of concentrated H_2_SO_4_ and 20 mL of HNO_3_ to measure As in rice and vegetables, separately. A gentle boiling digestion was performed using an electric heater after an overnight reaction. Once again, 5 mL of concentrated HNO_3_ was added to nearly 10 mL of digested brown solution for repeated digestion. When the digestion was still not complete after 2–3 additional HNO_3_ digestions, concentrated HClO_4_ was added (2 mL) for further digestion. However, 3–4 h was needed for the entire digestion process. The digest was transferred to a 25 mL measuring flask and spiked with 2.5 mL of a sulfourea solution (50 g/L), and the volume was made with double-deionized water. The As level in hair samples was assessed with a hydrogen generation–atomic fluorescence spectrometer (AFS-820, Beijing Titan Instruments Co., Beijing, China) after the samples were digested with a 1:4 mixture of HClO_4_ and HNO_3_ based on the procedure used by Liu et al. [37]. The samples were digested, and then 2% HCl was used to dissolve the remnants. All the reagents used were of an analytical grade or better. Tomato leaves (NIST 1573a) and Montana I soil (2710a) were used as certified reference materials during the quality assurance process. The range of values found for tomato leaves (NIST 1573a) was 0.109 μg/g to 0.120 μg/g against the certified value 0.112 ± 0.004 μg/g, and that for Montana I soil (2710a) was 608 to 626 μg/g against the certified value 636 ± 38 μg/g.

### 2.5. Statistical Analysis

Prior to analysis, collected data were encoded, entered into a Microsoft Excel 2019 spreadsheet, and double-checked for mistakes. SPSS 20.0 was used to analyze the data. Cross-tabulation in Excel was used to calculate descriptive statistics such as percentages and frequencies [20]. Mean, median, and standard deviation (SD) has been used to categorize farmers into low, medium, and high groups [20]. The Pearson correlation coefficient (r) was employed to analyze the correlation between dependent and independent variables [53]. This study included a stepwise multiple regression analysis to determine the socioeconomic parameters influencing perception in the research area [54]. Path analysis was carried out to determine independent variables’ influence and path effect on farmers’ perception [55]. Using R Statistics Software version 3.5.3, 2-way analysis of variance (ANOVA) and a least-significant-differences (LSD) test were conducted. Principle component analysis (PCA) was carried out using Minitab 18 statistical software [56].

## 3. Results and Discussion

### 3.1. Farmers’ Socioeconomic Characteristics

The summary of farmers’ socioeconomic characteristics is presented in Table S1 (Supplementary Materials). Almost two-thirds of the participants were in the middle-aged to old-age group, while 34% were in the young age group in this study. The rural youths’ paradigm shift is clearly articulated in terms other than agriculture [50]. Education is the process by which desired changes in human behavior take place. It is primarily supposed that a higher level of education should influence farmers to be aware of and critically evaluate the consequences of As-contaminated groundwater irrigation. Two-thirds of the respondents (66%) and slightly over 50% of their family members had primary and low to medium education, respectively, while 26% of participants passed secondary to above secondary classes. It could be seen that only 8% of respondents and 22% of family members were illiterate. Fewer than half (42%) of participants had small families, while 31% had large families. On the other hand, the knowledge status of the respondents showed that no less than 50% of farmers lack adequate knowledge of As and its impact on rice and vegetable cultivation with contaminated groundwater, while 34% possess high knowledge. All the participants in the study area had basic knowledge regarding groundwater contamination with As used for drinking water due to substantial awareness-building circulation from government and nongovernmental organizations in recent decades. However, the knowledge differences were created with an advanced aspect regarding crop contamination due to As-elevated groundwater irrigation. Family size also influences farmers’ perception of groundwater irrigation. More than half (58%) of the farmers had small farm holdings, 29% had medium farm holding, and only 4% had large (3.01-6.00 ha) farm holdings, which represents collective possession from own and others’ land in *borga*. Farmers with larger farms are predicted to be more eager to convert their land to irrigated fields to minimize their loss rather than keeping the land barren [50]. The results also revealed that farm size largely determined the annual income of the participants. Nearly 60% of the respondents had very low to medium incomes mainly derived from agriculture, particularly rice and vegetables. Of the rest, 19% had high and 20% had very high annual incomes from some business in addition to agriculture.

Cosmopoliteness influences farmers’ perception, since it enables them to be introduced to the latest technologies by exploring neighboring localities, towns, and abroad. Nearly half (48%) of the participants had low cosmopoliteness, followed by 32% with high cosmopoliteness. Similar to cosmopoliteness, the distribution of farmers based on information sources exposure showed that fewer than half (48%) of the participants had a low level of information sources exposure, followed by 52% with medium to high levels who have the latest agriculture information. The farmers’ educational status influenced their exposure to information sources. In addition, the information technology revolution had a profound impact on the farming communities.

All the farmers in this study had active participation in agricultural and farm management activities; however, they were categorized based on their extent of involvement. More direct participation in farming enhances actual field-based knowledge and experience and increases farm productivity due to the close observation and management possibility. More than half (57%) of the participants had medium to high direct participation in farming in their crop production, and the rest required some support from others for cultivation activities. Opinionatedness allows a farmer to exercise leadership capacity for the fellow crop growers regarding several decision-making processes, including crop variety selection, irrigation management, and intercultural operations. Nearly 50% of participants had low opinionatedness, while 27% had medium and 24% had high opinionatedness. Regarding agricultural credit use, almost half (49%) of the farmers did not use any credit; only 7% had low use, while 22% had medium to high use of credit for rice and vegetable production. Different banks, NGOs, cooperative organizations, and businessmen provide the credit. Although credit is presumed to be financial support for the initial period, the higher interest rates finally catch most farmers in a trap.

The organizational participation factor based on farmers’ distribution depicts that approximately half (49%) of the participants had low, one-third had high, and 18% had medium participation with different organizations. Organizational participation facilitates social networks to promote information flow, which stimulates farmers’ perceptions and decision making on agricultural management [57,58,59]. Farmers’ innovativeness in the adoption of As-mitigation irrigation management and other practices in the study area were evaluated. The results show that almost half (49%) of the respondents has no innovativeness, followed by 26% who have a medium level and 25% who have a high level. Ownership of agricultural machinery largely determines freedom of production management, especially irrigation practices with a specific strategy. The respondents mainly had similar agricultural machinery, with 43% and 31% possessing a medium and higher number of irrigation management tools, respectively. Table S1 (Supplementary Materials) also demonstrates that almost one-third of farmers had individual low-, medium-, and high-risk orientations. Those who had higher educational status, information sources used, and high organizational participation had a higher level of risk orientation [50]. In addition to the above, this study revealed that higher ownership of FPM also influences farmers’ risk orientation. However, this psychological character influenced farmers’ perception and adoption of the As-mitigating strategy.

### 3.2. Farmers’ Perception

According to McGraw-Hill [60], perception is the process by which sensory stimuli are registered as meaningful experiences, while Epstein et al. [61] understand perception as the way of dispersing stimulation through structured experiences. Perceptions are more sophisticated constructs made up of simple pieces connected by association and are therefore more susceptible to the influence of learning. Though the senses of taste, hearing, touch, and smell have all been investigated, vision has garnered the most interest. Perception is the process of becoming aware of or comprehending sensory information in psychology, philosophy, and cognitive science [60]. Table 1 demonstrates that 25% of the farmers possess good perception in the study area regarding As contamination in rice and vegetables due to contaminated groundwater irrigation as well as the drivers of irrigating As-elevated groundwater and possible mitigation strategies for and health impact of As. On the other hand, 36% demonstrated moderate perception, while 39% demonstrated poor perception. After a comprehensive assessment of farmers’ awareness regarding As in drinking water and foods, Mishra et al. (2021) reported that Bangladeshi farmers have comparatively high awareness regarding As in drinking water rather than in the foods they consume.

A total of 43 statements in seven categories were administered to obtain a detailed understanding of farmers’ perceptions (Table S2 in Supplementary Materials). All the farmers responded to each of the statements from their learned experiences. The following seven sections provide a brief overview.

#### 3.2.1. Perception on As-Contaminated Groundwater (AsW) and As-Free Water (AsFW) Use

Nearly two-thirds (62%) of the respondents strongly agreed and one-fourth agreed that no AsW means no rice/vegetable cultivation (Table S2 in Supplementary Materials). They opined that AsW is available throughout the year for crop cultivation in their locality, while AsFW is seasonal. Apart from this, an overwhelming (89%) of respondents still debated not using the AsFW in their fields. This might be because although they are aware of the drinking water As contamination, the majority of them still lack proper knowledge regarding possible crop contamination with As. On the other hand, only 19% of farmers believe in the possibility of rice and vegetable cultivation with AsFW. The explanation for such a stance is that they possess comparatively larger farm holdings with adequate irrigation management tools.

#### 3.2.2. Drivers for Irrigating AsW

Easy accessibility is the prime cause for AsW use and was unequivocally declared by all the participants in this study. Nearly 98% of the respondents claimed that they prefer irrigating their crop fields with some shareholders to reduce the production cost. This prevalent scenario of field irrigation practice threatens the choosy irrigation management in this study area. The scarcity of AsFW (e.g., surface water), particularly during the winter season, compels them to go for groundwater irrigation. Another reason for using AsW is the water-saving purpose of AsFW for household use, as reported by 26% of the respondents. Only 3% of the farmers are self-sufficient enough to irrigate with their own pump and manage irrigation as per their choice. 

#### 3.2.3. Effect of AsW Irrigation on Crop Fields

While demonstrating the impact of AsW irrigation on crop fields from their experiences, two-thirds of the farmers remained undecided whether the AsW led to add additional As in their crop fields or not, although the rest one-third believed in As addition. Similarly, four-fifths of the farmers were undecided regarding the fertility loss of their crop fields with As incorporation due to groundwater irrigation. On the other hand, slightly over 50% of the participants observed that their irrigation channel became red, 40% reported yield loss near the channel, and the land became hard. 

#### 3.2.4. Effect of AsW Irrigation on Rice & Vegetables

Only 19% of the respondents believe in As accumulation in rice and vegetables upon As-contaminated groundwater application. The levels of education, organizational participation, information source exposure, and cosmopoliteness enhanced their knowledge regarding this issue and influenced their perception. More than 95% of farmers were undecided about the other parameters such as the impact on tillering, influence on plants’ height, uniformity of flowering, plant growth and grain maturity, grains’ filling percentage, and yield reduction. However, only 2–4 percent of participants agree with those advanced symptoms. 

#### 3.2.5. Impact of Fertilizers and Pesticides on As Addition

Application of pesticides [62] and fertilizers, especially phosphate fertilizer [63], may escalate As levels in crop fields. Almost all the respondents were undecided, since they did not have such information from any media or social networking. 

#### 3.2.6. Health Impact

From their knowledge of groundwater As contamination and knowledge about the As-related health impact from drinking water exposure, 7% of the farmers agreed and 35% highly agreed with possible As transfer to the human body due to As-elevated rice and vegetable consumption. However, more than 50% of the respondents remained undecided. Similarly, 45% of the participants perceived As possibly causing cancers, while 39% agreed about the development of skin lesions.

#### 3.2.7. Farmers’ Practiced As-Mitigation Strategy

Nearly one-third of farmers perceive that alternate wetting and drying (AWD) and surface water irrigation can reduce As accumulation in rice and vegetables. Seven% of the participants believe that raised bed rice cultivation would limit As loading in rice grains. A very insignificant part (1–2%) of the participants perceive that fertilizer management, such as supplementing with more urea, MoP, gypsum, zinc sulphate, and cow dung, and using intercultural operations, such as mulching in vegetable fields and spreading ash, would limit As accumulation.

### 3.3. Correlations

Correlation coefficients between the independent and dependent variables has been estimated in Table S3 (in Supplementary Materials), and Table S4 (in Supplementary Materials) shows the correlation matrix representing the overall interaction between the variables. According to Table S3 (Supplementary Materials), among the socioeconomic characteristics, farmers’ age, annual income, family education, family size, farm size, and agricultural credit use were nonsignificant. In contrast, farmers’ age and family size were negatively correlated with their perception of As-elevated groundwater irrigation for rice and vegetable production. The studies by Alam [64] and Kabir [65] revealed a negative correlation of family size with perception, while Majlish [66] reported a nonsignificant correlation. Afique [67], Pal [68], and Adeola [69] revealed that farm size had no discernible effect on farmers’ perception. Friedler et al. [70] argued that there was no correlation between the age or income of farmers and their perception. Islam [71] observed no association between farmers’ utilization of credit and their perception.

On the other hand, farmers’ education, knowledge, information sources, direct participation in farming, cosmopoliteness, opinionatedness, innovativeness, risk orientation, farm power and machinery (FPM), and organizational participation were positively significant with perception at a 1% significance level (shown in Table S3 in Supplementary Materials). Pal [68] revealed that farmers’ education positively correlates with their perception. Kabir and Rainis [69] and Adeola [72] also found that education significantly affects farmers’ perceptions in Nigeria and Bangladesh, respectively. Individuals with higher education levels usually perceive risks and understand mitigation necessity in a very advanced way [73]. In their survey in Gujarat Province in India, Kumar and Popat [20] exposed that knowledge, a psychological characteristic of the farmer, had a significant positive association with farmers’ perception. A study by Adeola [69] reported similar findings in Nigeria. Farmers’ information sources can play a crucial role in building positive or negative perceptions of any phenomenon. Rezaei et al. [74] found a significant relationship between farmers’ exposure to information sources and their perception. Similarly, a study by Zhou et al. [75] with 278 mining farmers in Daye City, Hubei Province, demonstrated that knowledge and information have significant positive effects on perception. Farmers’ engagement in farming activities helps determine their decision-making capacity in any circumstance [76,77]. Therefore, direct farming participation had a significant relationship with farmers’ perceptions [78]. Islam [71] revealed a significant positive correlation between farmers’ perception and annual income.

Regarding the association between farmers’ ownership of FPM and their perception, Mottaleb et al. [79] in their study of the water markets in Bangladesh, demonstrate that irrigation pump ownership largely determines farmers’ perception. However, Mottaleb et al. concluded that since the irrigation system in Bangladesh is mainly based on pumping underground water, pump ownership significantly influences the structure and choice of irrigation practices. Regarding the relationship between organizational participation and perception, Keshavarz and Karami [80] reported that membership in social organizations positively influences farmers’ perceptions. Membership in formal or informal organizations helps farmers obtain benefits and social support [81]. Segnestam [82] argued that organizational participation helps disseminate innovation and develops mutual trust among the farmers, which eventually shapes farmers’ perceptions. While studying cosmopoliteness, Alam [64] noted a significant positive association between farmers’ cosmopoliteness and their perceptions. According to Hamid [83], there is a significant relationship between cosmopoliteness and farmers’ use of the recommended level of plant-protection practices. Farmers’ opinionatedness and perception were found to have a significant positive association in the study by Islam [71]. Londhe et al. [84] discovered a substantial positive relationship between perception and participants’ risk orientation and innovativeness. The study by Rekha and Ambujam [50] in Tamil Nadu, India, about farmers’ perception of contaminated water irrigation revealed a significant positive correlation between farmers’ perception and their educational status, information sources, annual income, farm size, risk orientation, and innovativeness.

### 3.4. Regression Esults

Predictor variables (independent variables) that explain farmers’ perceptions (the dependent variable) were determined using a stepwise multiple regression analysis. Table 2 illustrates the findings of stepwise regression. The total variance explained by the five independent variables is 0.884 (R = 0.889, R^2^ = 0. 884), as seen in Table 2. Of the total variance, participants’ knowledge explained 74.6%, direct participation in farming 8.2%, information sources 4.5%, participant education 0.7%, and organizational participation 0.8%. The F value for participants’ knowledge, direct participation in farming, and information sources is significant at the 0.1% level, while the F value for for participants’ education and organizational participation is significant at the 5% level. This means that the five recognized predictor variables account for 88% of the variance in the dependent variables.

### 3.5. Path Analysis

**Figure 1:**
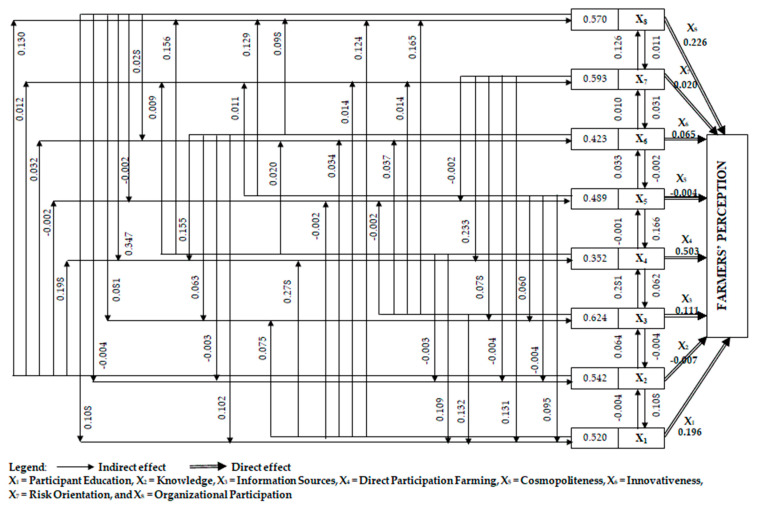
Path analysis.

With the path analysis, the total effects are broken down into indirect and direct effects on certain independent variables. Direct participation in farming presents the highest positive total effect (0.855) and direct effect (0.503), whereas information sources show the highest positive indirect effect (0.624) (Figure 1). Organizational participation (0.796, 0.226) and participant education (0.716, 0.196) represent the second- and third-highest total and positive direct effects, respectively, both with positive impact (Table S5 in Supplementary Materials). Risk orientation (0.593) ranked second and organizational participation (0.570) ranked third in terms of positive indirect effect. Out of the eight independent variables, four [participant education (X1), knowledge (X2), information sources (X3), and cosmopoliteness (X5)] have the highest indirect effect on farmers’ perceptions of transformation through direct participation in farming and organizational participation. On the other hand, another three [innovativeness (X6), risk orientation (X7), and organizational participation (X8)] have the highest indirect effect through direct participation in farming and participant education, which are depicted in Table S5 (Supplementary Materials). However, path analysis revealed that just a few variables directly impacted farmers’ perception levels, while interconnected variables were principally involved in the effect of several variables on farmers’ perceptions.

### 3.6. Arsenic Content in Collected Samples

The study revealed As content in irrigation water (ranges 0.108–0.356, 0.111–0.338, 0.110–0.371, 0.041–0.364, and 0.065–0.356), soils (ranges 15.645–30.675, 14.325–29.612, 16.327–32.1, 11.895–32.667, and 11.375–32.262), vegetables (on dry weight basis) (ranges 0.83–3.56, 0.26–3.25, 0.45–3.8, 0.21–3.9, and 0.23–3.84), rice grains (on dry weight basis) (ranges 0.192–0.75, 0.22–0.69, 0.18–0.89, 0.09–0.86, and 0.117–0.74), and farmers’ scalp hair (ranges 0.34–2.21, 0.4–2.36, 0.42–2.38, 0.32–2.44, and 0.35–2.17) for Sadar, Faridganj, Matlab north, Kachua, and Hajiganj, respectively. Table 3 demonstrates the probability level of As content in all five items. Arsenic in irrigation water is significantly different at a 1% probability level in the study sites.

The lowest As content in irrigation water is revealed is from Matlab north, while the highest is found in Faridganj. Similarly, As levels in the study sites’ soil is significantly different (*p* ≤ 0.01), where Matlab north and Hajiganj’s soil contains statistically similar As to Sadar and Faridganj. Significantly (*p* ≤ 0.05) higher As is found in grains from Faridganj and Hajiganj compared with grains from Kachua, Matlab north, and Sadar. In contrast, the lowest and highest grain As is recorded in Matlab north and Faridganj, respectively. Vegetable As in all the five study areas differs significantly at a 1% probability level. The vegetable As level from Hajiganj is pretty close to that of Faridganj, and the same is true for Sadar, while the level from Kachua is very close. Similar to grain As content, the lowest and highest vegetable As is recorded in Matlab north and Faridganj, respectively. There is a significant correlation between soil As concentrations and the accumulation of As in rice grains [90,91]. Comparable patterns of arsenic uptake in plants cultivated in regions with high soil concentrations of As and irrigated by arsenic-rich groundwater have been discovered [92,93]. One of the most important methods for calculating the rate of chronic As exposure is to evaluate the amount of As in scalp hair [94,95]. As is commonly found in hair at background levels of 0.08–0.250 mg/kg [96], 1 mg As per kg hair has been unanimously established as the toxicity indicator [96,97,98]. Faridganj and Hajiganj have been found to have significantly (*p* ≤ 0.001) higher and closely resemble hair As concentration. Again, hair As levels observed from Matlab north and Kachua are also statistically similar. Hair As content of Sadar is also in immediate proximity that of Matlab north and Kachua. At all five locations, the mean As concentration in vegetables and irrigation water is much higher compared to the permissible limit [3,4], while the As level in soil is higher than the As level on a global scale but below the FAO-proposed limit for agriculture [85,86]. Except for Matlab north, grain As content surpassed the safe limit [99] at all places. On the other hand, scalp hair As is recorded as being above the toxicity limit for four locations except Sadar but above the background value. Results suggest significant As transfer from irrigation water to rice and vegetables and subsequent body loading. This result is in consensus with the finding of Joardar et al. [95], who reported As accumulation in scalp hairs of a number of arsenicosis patients due to As-contaminated rice and vegetables consumption, although the accumulation pattern was diverse.

### 3.7. Principal Component Analysis (PCA)

**Figure 2:**
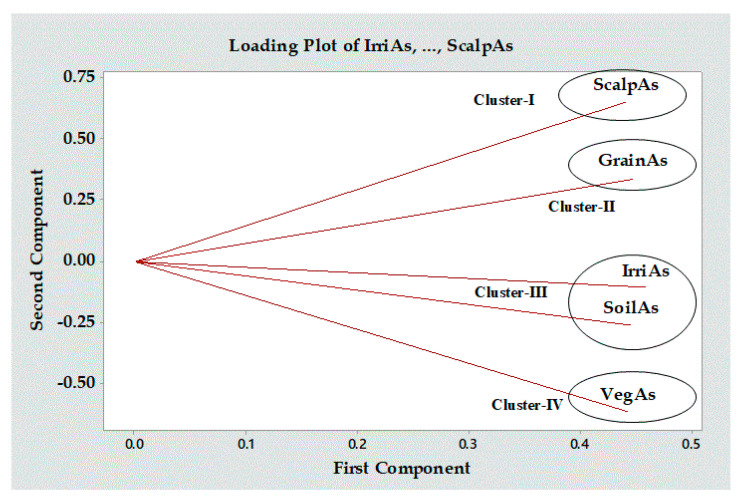
Principle component analysis (PCA). IrriAs = irrigaiton water As; SoilAs = soil As; GrainAs = grain As; VegAs = vegetables A; ScalpAs = scalp hair As.

Figure 2 depicts four unique clusters that are produced by the varying lengths of the eigenvectors. Correlations between items are represented by the angle between eigenvectors, and the length of each eigenvector is proportional to the variance of the corresponding data item. Hair As, grain As, water As, soil As, and vegetable As are all examples of factors that fall into one of the five categories denoted by Clusters (I), (II), (III), and (IV). Parameters with identical values are observed to cluster together in Figure 2. This divergence can be explained by the fact that the As in irrigation water and soil (Cluster III) contributes to a similar variance, while the As in scalp hair (Cluster I), grain (Cluster II), and vegetables (Cluster IV) does not. Lengthwise, Cluster II was the lowest, and Cluster IV was the highest, suggesting the lowest and highest variations, respectively. It is clear that there is a strong relationship between categories (I) and (II) among the four options. Table S6 (in the Supplementary Materials) displays the PCA results for As concentration of various parameters. Table S6 shows that the first principal component (PC) has an eigenvalue greater than 1, indicating that it adequately describes the variances. As for irrigation water (0.458), grain (0.448), and soil (0.446), these three factors account for the vast majority (92.5%) of the total variance explained by the first PC (Table S6). Values highlighted in bold in the Table are particularly relevant for understanding the PC, as a higher numerical value denotes a more substantial contribution. Thus, the PC1 loading values were largely influenced by the parameters of irrigation water As, grain As, and soil As.

## 4. Conclusions

The most concerning health issue in naturally As-endemic regions is the high concentration of As in groundwater and its subsequent transfer to the human body via rice and vegetable consumption. The level of farmers’ perception about the source of As contamination, As-induced ailment and symptoms, and potential measures to minimize crop loading with As was investigated in this study. This current study revealed that only one-fourth of the farmers gave a positive message with good perceptions regarding the As-contamination scenario in rice and vegetables, and even the additional 36% of people with moderate perceptions give cause for optimism. Although 10 of farmers’ socioeconomic characteristics were positively significant (*p* ≤ 0.01) and likely to influence their perceptions in a positive direction, distinctive emphasis should be given to participants’ knowledge, direct participation in farming, information sources used, education, and organizational participation, 5 socioeconomic factors explain 88% variances in perception. Path analysis depicts that direct participation in farming presents the highest positive total effect (0.855) and direct effect (0.503), whereas information sources show the highest positive indirect effect (0.624). Arsenic in scalp hairs, vegetables, rice, and irrigation water exceeded the permissible limit. Statistically significant at the 5%, 5%, 0.1%, 1%, and 1% probability levels, mean As content in scalp hairs, rice, vegetables, soils, and irrigation water, respectively, was observed in all the study sites. The first principal component (PC1) explains 92.5% of the variation, and the irrigation water, grain, and soil As are the dominating parameters. This study clearly shows that As perception is not widespread among the farmers, the primary stakeholder, at this time, although significant As contamination and transfer to crops is evident. While most participants had at least some perception of the As problem in irrigation water and its uptake by rice and vegetables, their knowledge gap is notably prominent regarding the mitigation measures available to prevent contamination. These are crucial aspects in formulating policies in all the As-endemic nations. This study assessed a preliminary context, that is, farmers’ perception of As-contaminated groundwater irrigation for rice and vegetable cultivation. We recommend further studies on farmers’ attitudes towards adopting As-mitigation strategies in an interdisciplinary context, emphasizing the socioeconomic status revealed in this study influencing farmers’ perception.

## Figures and Tables

**Table 1:** Farmers’ perception on arsenic-contaminated groundwater irrigation for rice and vegetables production (N = 200).

Category	Percent	Mean	Standard Deviation
Poor perception (129–136)	39		
Moderate perception (137–155)	36	146.6	14.16
Good perception (157–178)	25		
Total	100		

**Table 2:** Regression of the estimated perception on the independent variables (N = 200).

Variables	R	R Square	Adjusted R Square	Std. Error of the Estimate	R Square Change	F Change	Sig. F Change
Participants/ knowledge	0.865	0.748	0.746	7.140	0.748	291.373	0.000
Direct participation in farming	0.911	0.830	0.826	5.899	0.082	46.587	0.000
Information sources	0.935	0.875	0.871	5.089	0.045	34.297	0.000
Participant education	0.939	0.882	0.877	4.973	0.007	5.533	0.021
Organizational participation	0.943	0.889	0.884	4.832	0.008	6.638	0.012

**Table 3:** Comparison of As concentration in different components collected from five (05) different location of Chandpur districts of Bangladesh.

Locations	As in Irrigation Water (mg/L) (against Background Value 0.1 mg/L by FAO and 0.01 mg/L by WHO [3,4])	As in Soil (mg/kg) (against Global Average 10 and FAO Limit 50 mg/kg [85,86])	As in Vegetable (mg/kg) (against Permissible Limit 0.5 to 1.0 mg/kg [37,87])	As in Grain (mg/kg) (against Permissible Limit 0.37 mg/kg [88])	As in Hair (mg/kg) (against Background Value 0.08–0.250 and Toxicity Indicator 1.0 mg/kg [89])
Hajiganj	0.227ab	21.90b	2.03ab	0.459a	1.24ab
Kachua	0.204bc	20.69c	1.82cd	0.418b	1.08c
Matlab north	0.192c	21.10b	1.61d	0.367c	1.00c
Faridganj	0.234a	23.00a	2.21a	0.472a	1.28a
Sadar	0.217b	23.08a	1.93c	0.399bc	0.96cd
LS	**	**	***	*	*
CV (%)	6.81	8.81	5.51	6.28	6.70
SE (±)	1.17	0.93	1.24	1.15	0.96

In columns, means followed by different letters are significantly different. LS, means level of significance; CV, means coefficient of variance; SE, means standard error; ***, means at 0.1% level of probability; **, means at 1% level of probability; and *, means at 5% level of probability.

## Data Availability

Data will be made available upon request. The data are not publicly available due to ethical issues.

## Supplementary Materials

The supplementary material published online alongside the manuscript. The following supporting information can be downloaded at: https://www.mdpi.com/article/10.3390/ijerph20064989/s1, Table S1. Farmers’ socioeconomic characteristics (N = 200); Table S2. Farmers’ perception scores on different parameters (N = 200); Table S3. Correlation coefficients (r) between farmers’ perception and their socioeconomic parameters; Table S4: correlation matrix representing overall interaction between the variables; Table S5. Decomposition of total effects into direct and indirect effect of independent variables on perception of farmers towards transformation (n = 200); Table S6. Principal components and their eigenvalue, %variance and cumulative (%).

## Appendix A

### Appendix A.1

Hajiganj is located at 23.2500° N 90.8500° E and the total land area is 189.90 km^2^; Kachua is located at 23.3500° N 90.8917° E, having a total land area of 235.82 km^2^; Matlab is north at 23.3500° N 90.7083° E, and total land area is 131.69 km²; Faridganj is at 23.1250° N 90.7486° E, and total land area is 231.56 km^2^; and Chandpur Sadar (Sadar) is situated at 23.2139° N 90.6361° E, with a total land area of 308.78 km^2^.

### Appendix A.2

An amount of 100 mL of groundwater was collected, where 5 mL of 2M hydrochloric acid was added immediately and filtered with 0.45-µ Millipore filters. Soil subsamples were collected directly from the upper 0–20 cm horizon of the standing crop fields with assembled sectional auger by making four holes at the corners of the grid of 20 m^2^ as per the IGCP 259 recommendations [99] (Fordyce et al., 2000). The soil samples were screened to pick out stones/gravel/residue and were air-dried and sieved twice through 2 mm and 0.149 mm mesh and then wrapped in kraft paper wrappers until analysis. Subsamples of standing rice and edible portions of vegetables were also collected directly from the same field samples and stored safely in polyethylene zip lock bag and then quickly shifted to the laboratory [100].

Like rice, the commercial cultivation of vegetables in the study area is a common practice. Therefore, vegetables cultivated simultaneously with the rice or after harvesting rice were collected and irrigated with the groundwater from the same source used for rice. The plant samples include rice (*Oryza sativa*) of different varieties, arum roots (*Colocasia esculenta*), beans (*Phaseolus vulgaris*), radish (*Raphanus sativus* L), potato (*Solanum tuberosum*), brinjal/eggplant (*Solanum melongena*), cauliflower (Brassica oleracea var. botrytis L), cabbage (*Brassica* oleracea var. capitata), and tomato (*Solanum lycopersicum*) as well as some leafy vegetables such as water spinach (*Ipomoea aquatic*) and red and stem amaranth (*Amaranthus* spp.).
