# Supplementary material for: Arsenic Elevated Groundwater Irrigation: Farmers’ Perception of Rice and Vegetable Contamination in a Naturally Arsenic Endemic Area
Source: Int J Environ Res Public Health. 2023 Mar 12;20(6):4989. doi: 10.3390/ijerph20064989 (PMC10049387; doi:10.3390/ijerph20064989)
Supplement: Supplementary file 1 [file ijerph-20-04989-s001.zip › ijerph-2216416-supplementary.pdf]

Supplementary materials

Table S1. Farmers′ socioeconomic characteristics (N=200)

Characteristics (Measuring units)	Percent	Mean	Standard Deviation	Characteristics (Measuring units)	Percent	Mean	Standard Deviation
**Age Groups in years**				**Participant Education**			
Young (18-37)	34			Illiterate (0)	8		
Middle aged (38-50)	36	42.63	11.1	Primary (1-5)	66	3.73	2.83
Old (51-70)	30			Secondary (6-10)	16		
**Family Size**				Above secondary (11 and above)	10		
Small (1-3)	42			**Family Education**			
Medium (4-5)	27	4.29	1.92	No Education	22		
Large (6-10)	31			Low Education	32	4.36	3.94
**Knowledge**				Medium Education	26		
Low knowledge (4-12)	52			High Education	20		
Medium knowledge (13-20)	14	16.05	8.49	**Farm Size**			
High knowledge (21-35)	34			Marginal farm size (0.04-0.20)	9		
**Information Source**				Small farm size (0.21-1.00)	58	1.03	0.98
Low cosmopoliteness (2-6)	48			Medium farm size (1.01-3.00)	29		
Medium cosmopoliteness (7-11)	20	18.68	12.98	Large farm size (3.01-6.00)	4		
High cosmopoliteness (12-17)	32			**Annual income (in 1000 bdt)**			
**Direct Participation in Farming**				Very low income (45-90)	21		
Low direct participation in farming (10-12)	43			Low income (91-162)	19		
Medium direct participation in farming (13-15)	34	13.68	2.59	Medium income (163-250)	21	247.6	185.73
High direct participation in farming (16-18)	23			High income (251-344)	19		
				Very high income (345-750)	20		
**Credit use (in 1000 bdt)**				**Cosmopoliteness**			
No credit use (0)	49			Low cosmopoliteness (2-6)	48		
Low credit use (0.01-10)	7			Medium cosmopoliteness (7-11)	20	8.02	4.31
Medium credit use (11-30)	22	20.41	27.86	High cosmopoliteness (12-17)	32		
High credit use (30-100)	22			**Innovativeness**			
**Opinionatedness**				No innovativeness (0)	49		
Low opinionatedness (2-5)	49			Low to medium innovativeness (1-5)	26	3.31	3.62
Medium opinionatedness (6-8)	27	6.42	3.26	Upper medium to high innovativeness (6-14)	25		
High opinionatedness (9-12)	24			**Farm power machinery ownership**			
**Risk orientation**				Low ownership (5-9)	26		
Low risk orientation (12-18)	34			Medium ownership (10-14)	43	12.56	4.04
Medium risk orientation (19-29)	34	26.53	10.31	High ownership (15-22)	31		

High risk orientation (30-47)	32	**Organizational participation**			
		Low organizational participation (4-10)	49		
		Medium organizational participation (11-18)	18	16.62	10.5
		High organizational participation (19-38)	33		

Table S2. Farmers' perception scores on different parameters (N=200)

**Sl No.**	**Parameters**	**Frequency**				
		**Strongly disagree**	**Disagree**	**Undecided**	**Agree**	**Strongly agree**
	**Perception on As-contaminated water (AsW) or As free water use (AsFW)**					
1	For me, no AsW means no rice/vegetable production	8	0	16	52	124
2	I can produce rice/vegetables without AsW	80	0	82	12	26
3	Why on earth would I use AsFW to produce vegetables?	8	0	6	178	8
4	Why on earth would I use AsW to produce rice/vegetable	0	0	190	6	4
5	AsW for rice/vegetable production is available throughout a year	0	0	2	68	130
6	AsFW for rice/vegetable production is available throughout a year	88	70	2	14	26
7	AsW for use in vegetable production is seasonal	98	86	2	0	14
8	AsFW for use in vegetable production is seasonal	0	4	20	66	110
	**Drivers of irrigating AsW**					
9	Easily accessible	0	0	0	68	132
10	I can irrigate with other shareholders of pumps	2	4	0	60	134
11	I pay little money to get AsW for use in my rice/vegetable production	4	2	0	62	132
12	Scarcity of AsFW	2	4	0	68	126
13	Decrease production cost	4	2	0	62	132
14	Saving AsFW for household use	4	74	66	16	40
	**Effect of AsW irrigation on crop fields**					
15	Crop fields contaminated with arsenic	0	0	128	2	70
16	Decreases soil fertility	0	0	162	16	22
17	Irrigation canals and rice/vegetables fields become red	0	0	96	26	78
18	Land become hard	0	0	118	12	70
19	Rice/vegetables yield near irrigation channel/STW is low	0	2	120	4	74

	**Effect of AsW Irrigation on rice & vegetables**					
20	Less tillering	0	0	190	2	8
21	Plants become shorter in height	0	0	196	2	2
22	Plant growth not uniform	0	0	198	2	0
23	Plants do not flower uniformly	0	0	198	0	2
24	Grains do not mature uniformly	0	0	196	4	0
25	More unfilled grains	0	0	198	2	0
26	Decrease rice yield	0	0	196	2	2
27	As may accumulate in rice/vegetables upon irrigating with AsW	0	0	162	26	12
	**Impact of fertilizers and pesticides on As addition**					
28	Application of pesticides may induce As in crop fields	0	0	198	2	0
29	Application of chemical fertilizers (especially TSP) add As in soils	0	0	198	2	0
	**Health impact**					
30	Consumption of contaminated rice/vegetables may transfer As to human body	0	0	116	14	70
31	As may cause cancers	0	0	110	22	68
32	Skin lesson	0	0	122	14	64
	**Farmers' practiced As mitigation strategy**					
33	irrigate and allow the field to dry (AWD)	0	0	130	12	58
34	Use stored groundwater for irrigation	0	0	194	4	2
35	Practice raised bed cultivation	0	0	186	10	4
36	Use surface water for irrigation	0	0	128	20	52
37	Apply more urea	0	0	194	2	4
38	Apply more MoP	0	0	196	2	2
39	Apply more gypsum fertilizer	0	0	198	2	0
40	Apply more zinc sulphate	0	0	198	2	0
41	Did mulching	0	0	198	2	0
42	Apply cow dung	0	0	198	2	0
43	Apply Ash	0	0	198	2	0

Table S3. Correlation coefficients (r) between farmers' perception and their socioeconomic parameters

**Independent**	**Dependent**	**Correlation coefficients (r)**
Age of the participant	Farmers' Perception	-0.022NS
Farmers education		0.716**
Family education		0.038NS
Annual income		0.165NS
Family size		-0.045NS
Knowledge		0.865**
Information sources		0.735**
Farm size		0.145NS
Direct participation farming		0.855**
Agricultural credit use		0.148NS
Cosmopoliteness		0.485**
Opinionatedness		0.512**
Innovativeness		0.488**
Risk orientation		0.613**
Farm power and machinery (FPM)		0.269**
Organizational participation		0.796**

** Correlation is significant at the 0.01 level (2-tailed)

* Correlation is significant at the 0.05 level (2-tailed)

Table S4: correlation matrix representing overall interaction between the variables

	X1	X2	X3	X4	X5	X6	X7	X8	X9	X10	X11	X12	X13	X14	X15	X16
X1	1															
X2	-.022	1														
X3	.716**	.009	1													
X4	.038	.380**	.304**	1												
X5	-.045	.878**	.009	.424**	1											
X6	.865**	-.121	.666**	.030	-.156	1										
X7	.735**	.061	.676**	.048	.085	.621**	1									
X8	.145	-.018	.179	-.037	-.014	.082**	.19	1								
X9	.855**	-.059	.554**	.045	-.101	.783**	.559**	.174	1							
X10	.148	.052	.243*	.211*	.13	.166	.149	.420**	.062	1						
X11	.485**	.038	.484**	.039	.032	.426**	.537**	.294**	.330**	.163	1					
X12	.512**	-.072	.542**	.115	-.052	.527**	.615**	.247*	.407**	.181	.476**	1				
X13	.488**	.011	.522**	.225*	.038	.395**	.565**	.199*	.308**	.125	.509**	.514**	1			
X14	.613**	.043	.670**	.125	.067	.606**	.700**	.042	.463**	.193	.527**	.567**	.473**	1		
X15	.269**	-.032	.228*	.084	-.037	.233*	.332**	.161	.235*	.259**	.107	.056	.230*	.231*	1	
X16	.796**	-.023	.550**	.000	-.062	.722**	.731**	.260**	.691**	.087	.570**	.562**	.435**	.557**	.196	1

** Correlation is significant at the 0.01 level (2-tailed).

* Correlation is significant at the 0.05 level (2-tailed).

Listwise N=100

Participation Characters	Symbol	Participation Characters	Symbol		
Perception	X1	Knowledge	X6	Cosmopoliteness	X11
Participant age	X2	Information sources	X7	Opinionatedness	X12
Participant education	X3	Farm size	X8	Innovativeness	X13
Family education	X4	Direct participation in farming	X9	Risk orientation	X14
Family size	X5	Credit use (1000 BDT)	X10	Ownership of farm power and machinery (FPM)	X15
				Organizational participation	X16

Table S5. Decomposition of total effects into direct and indirect effect of independent variables on perception of farmers towards transformation (n = 200)

Independent variable	Direct effect	Total indirect effect	Variable through which substantial indirect effects were channelized	
Participant education (X_1)	0.196	0.520	-0.004	Knowledge (X2)
			0.075	Information sources (X3)
			0.278	Direct participation in farming (X4)
			-0.002	Cosmopoliteness (X5)
			0.034	Innovativeness (X6)
			0.014	Risk orientation (X7)
			0.124	Organizational participation (X8)
Knowledge (X_2)	-0.007	0.542	0.108	Participant education (X_1)
			0.064	Information sources (X3)
			0.198	Direct participation in farming (X4)
			-0.002	Cosmopoliteness (X5)
			0.032	Innovativeness (X6)
			0.012	Risk orientation (X7)
			0.130	Organizational participation (X8)
Information sources (X_3)	0.111	0.624	0.132	Participant education (X_1)
			-0.004	Knowledge (X2)
			0.281	Direct participation in farming (X4)
			-0.002	Cosmopoliteness (X5)
			0.037	Innovativeness (X6)
			0.014	Risk orientation (X7)
			0.165	Organizational participation (X8)
Direct participation in farming (X_4)	0.503	0.352	0.109	Participant education (X_1)
			-0.003	Knowledge (X2)
			0.062	Information sources (X3)
			-0.001	Cosmopoliteness (X5)
			0.020	Innovativeness (X6)
			0.009	Risk orientation (X7)
			0.156	Organizational participation (X8)
Cosmopoliteness (X_5)	-0.004	0.489	0.095	Participant education (X_1)
			-0.004	Knowledge (X2)

			0.060	Information sources (X3)
			0.166	Direct participation in farming (X4)
			0.033	Innovativeness (X6)
			0.011	Risk orientation (X7)
			0.129	Organizational participation (X8)
Innovativeness (X_6)	0.065	0.423	0.102	Participant education (X_1)
			-0.003	Knowledge (X2)
			0.063	Information sources (X3)
			0.155	Direct participation in farming (X4)
			-0.002	Cosmopoliteness (X5)
			0.010	Risk orientation (X7)
			0.098	Organizational participation (X8)
Risk orientation (X_7)	0.020	0.593	0.131	Participant education (X_1)
			-0.004	Knowledge (X2)
			0.078	Information sources (X3)
			0.233	Direct participation in farming (X4)
			-0.002	Cosmopoliteness (X5)
			0.031	Innovativeness (X6)
			0.126	Organizational participation (X8)
Organizational participation (X_8)	0.226	0.570	0.108	Participant education (X_1)
			-0.004	Knowledge (X2)
			0.081	Information sources (X3)
			0.347	Direct participation in farming (X4)
			-0.002	Cosmopoliteness (X5)
			0.028	Innovativeness (X6)
			0.011	Risk orientation (X7)

Table S6. Principal components and their eigenvalue, %variance and cumulative (%)

	PC1	PC2	PC3	PC4	PC5
Eigenvalue	4.6271	0.1622	0.1069	0.0736	0.0302
Proportion	0.925	0.032	0.021	0.015	0.006
Cumulative	0.925	0.958	0.979	0.994	1.000
Variable					
IrriAs	**0.458**	-0.109	-0.137	0.137	-0.861
SoilAs	**0.446**	-0.261	0.722	0.405	0.220
VegAs	0.443	-0.616	-0.298	-0.507	0.281
GrainAs	**0.448**	0.337	-0.547	0.504	0.363
ScalpAs	0.441	0.653	0.268	-0.554	0.022
